# Genomic Adaptation of *Saccharomyces* Species to Industrial Environments

**DOI:** 10.3389/fgene.2020.00916

**Published:** 2020-08-27

**Authors:** Konstantina Giannakou, Mark Cotterrell, Daniela Delneri

**Affiliations:** ^1^Manchester Institute of Biotechnology, Faculty of Biology, Medicine and Health, The University of Manchester, Manchester, United Kingdom; ^2^Cloudwater Brew Co., Manchester, United Kingdom

**Keywords:** fermentation, *Saccharomyces*, adaptation, diversity, evolution

## Abstract

The budding yeast has been extensively studied for its physiological performance in fermentative environments and, due to its remarkable plasticity, is used in numerous industrial applications like in brewing, baking and wine fermentations. Furthermore, thanks to its small and relatively simple eukaryotic genome, the molecular mechanisms behind its evolution and domestication are more easily explored. Considerable work has been directed into examining the industrial adaptation processes that shaped the genotypes of species and hybrids belonging to the *Saccharomyces* group, specifically in relation to beverage fermentation performances. A variety of genetic mechanisms are responsible for the yeast response to stress conditions, such as genome duplication, chromosomal re-arrangements, hybridization and horizontal gene transfer, and these genetic alterations are also contributing to the diversity in the *Saccharomyces* industrial strains. Here, we review the recent genetic and evolutionary studies exploring domestication and biodiversity of yeast strains.

## Yeast Evolution in Industrial Settings

Beer brewing and winemaking have been rapidly changing over the years involving the development of several fermentation protocols and the use of starter cultures. In the past, these processes were mainly occurring naturally. For example, grape juice and fresh hoppy wort were exposed to open air microorganisms to spontaneously ferment wine and beer.

While this natural process still find application in some beers (traditional lambic style, Spitaels et al., 2014) and specific type of wines (Walker, 2014; Chen et al., 2020), commercial products now largely employ the usage of starter cultures. The first pure yeast starter was created and used in beer production back in 1880s by E. C. Hansen from the Carlsberg laboratory in Denmark. In 1890s, also the first inoculation of a grape must with a yeast starter was performed. These practices became more common and *Saccharomyces cerevisiae* starters are primarily being used in wine and beer fermentations, to facilitate the consistency of fermented beverages resulting in products with stable characteristics, aromas and flavors as well as ensuring rapid fermentation times (Valero et al., 2007).

Yeast species belonging to the *Saccharomyces* genus have been extensively used in fermentation, and throughout the years the ability to ferment has evolved from the exposure to stressful conditions (Gallone et al., 2016). *Saccharomyces* “make-accumulate-tolerate-consume” strategy enables fast growth during anaerobic conditions, maximizes ethanol and flavor metabolites production together with preventing growth of antagonistic microbes by creating a hostile environment for them to survive (Piškur et al., 2006; Goold et al., 2017). During wine and beer fermentation yeasts are exposed to numerous stresses such as high osmotic pressure, oxidative stress, temperature shifts, low oxygen availability, CO_2_ accumulation, nutrient restraint and high ethanol concentration (Legras et al., 2018). Typically, *Saccharomyces spp.* expresses its fermentative ability either in mixture of high sugar environments such as brewing wort and grape juice or even in hydrolyzed lactose in fermented milk. As a result of this environmental variation, yeast strains diversified extensively in industrial settings and became adapted to the production of specific beverages (Table 1).

**TABLE 1:** *Saccharomyces* spp. in production of alcoholic beverages.

Environment	Microorganism	Product	Stresses induced	References	Favorable characteristic
Brewing	*S. pastorianus*	Lager style beers	Cold fermentation temperature (7–10°C), maltose utilization, oxygen depletion	Hill, 2015	Clean “non-fruity” taste
Brewing	*S. cerevisiae*	Ale style beer	Maltose utilization, oxygen depletion	Hill, 2015	Fruity and aromatic taste
Brewing	*S. cerevisiae var. diastaticus*	German wheat beers	Starch utilization	Meier-Dörnberg et al., 2018	Phenolic flavor
Winemaking	*S. bayanus*	Chardonnay wines	High sugar content, high alcohol tolerance	Eglinton et al., 2000; Kelly et al., 2018	Application in cool climate vineyards
Winemaking	*Flor yeast*	Spanish sherry wines	O_2_ presence, alcohol as the main carbon source	Jackson, 2014	Acetaldehyde
Winemaking	*S. cerevisiae S. bayanus*	Champagne	High ethanol tolerance	Martínez-Rodríguez et al., 2001	Foaming
Cider	*S. bayanus*	Ice-cider	Hyperosmotic stress, low pH, 15°C fermentation	Pando Bedriñana et al., 2017	Minimum residual sugars, 10% (v/v) final alcohol content
Sake	*S. cerevisiae*	Sake	High sugar content, high ethanol content	Katou et al., 2009	Isoamyl acetate, ethyl caproate
Distilling	*S. cerevisiae*	Whisky	40°C fermentation, high alcohol content	Walker et al., 2012	Dry finish, flavor consistency
Distilling	*S. cerevisiae*	Tequila	High fructose and ethanol content	Aldrete-Tapia et al., 2018	Reduced fermentation times

The extent of these adaptations can be easily detected in *S. cerevisiae* and *S. pastorianus*, the workhorses of beer fermentation, that have shown wide phenotypic variation to different beer fermentation conditions. These species have evolved so that their unique complexity and diversity can generate different beer related products. Some characteristics of industrial isolates are high consumption rate of complex sugars like maltose and maltotriose, enhanced tolerance to hyperosmotic stress, high ethanol production and simultaneous repression of undesired metabolites such as beer off-flavors (Gibson et al., 2013; Steensels et al., 2014; Hill, 2015; Gallone et al., 2018). Normally, brewing strains are able to ferment adequately a 12–14 P wort and produce 5–6% ethanol (v/v). However, some yeasts are suitable for “high gravity” brewing (i.e., high amount of total sugars diluted in water) as they are able to utilize the elevated sugar content and tolerate the higher ethanol concentrations. In high gravity brewing, highly concentrated wort is fermented to beer and then diluted to the desired ethanol concentration. It is a sustainable approach for increasing brewery yields, reduce production costs, and produce a variety of different products with higher or lower alcohol levels (Pátková et al., 2000; Piddocke et al., 2009; Caspeta et al., 2019). Wort gravity also affects the final beer flavor and the formation of volatiles and thus not all yeast strains are suitable for this fermentation (Piddocke et al., 2009; Lei et al., 2012). Industrial isolates from different sources have phenotypes associated with adaptation to that specific source. For example, rapid maltose utilization and fermentation is found in baker’s yeast (Bell et al., 2001) and higher ethanol production rates in sake and wine yeast (Uebayashi et al., 2018), however, the same ability varies significantly in non-industrial yeast strains.

## Mechanisms to Induce Genetic Variation in Industrial Strains

The environmental discontinuity has facilitated genetic diversification and phenotypic plasticity of yeast strains. Molecular patterns of domestication have now been explored in industrial yeasts, and significant variation have been shown among *Saccharomyces* beer, wine, sake and cider strains. *S. cerevisiae* brewing strains have shown remarkable population differentiation and are polyphyletic deriving from different geographical beer clades such as German, Belgian, and British ale. On the contrary wine, sake, and bread yeast have not shown much phenotypic diversity and are monophyletic (Almeida et al., 2015; Gonçalves et al., 2016). Population genomic studies of hundreds of *Saccharomyces* yeast strains reveal a remarkable level of variation in recombination rates and patterns even across very closely related lineages which can potentially translate in different phenotypic characteristics (Liti et al., 2009; Schacherer et al., 2009; Almeida et al., 2015). Advances in sequencing technologies have helped identification of yeast’s genetic traits underpinning different phenotypes from a plethora of environments (Sniegowski, 1999; Cromie et al., 2013; David et al., 2014). For instance, High- Throughput Sequencing approaches facilitated the understanding of the grape microbiome in different environmental conditions. In grapes and berries the identified microbiome included species belonging to the family of *Dothioraceae*, *Pleosporaceae*, *Saccharomycodaceae*, *Enterobacteriales*, *Pseudomonadales*, *Bacillales*, and *Rhodospirillales* and differences in field origin were examined for its relevance to wine fermentation and production of flavor metabolites in Cannonau wine from Sardinia (Mezzasalma et al., 2017). Sequencing of polyploidy beer strains revealed a common genetic ancestry with wine strains from European and Asian lineages. Polyploidization facilitated the gain or loss of genetic variation related with brewing characteristics and also indicates the usage of co-cultures has been employed in fermented beverages (Fay et al., 2019).

Several mechanisms that accelerate evolution in environmental challenges and stressful conditions have been studied. Yeast adapt to new environments via smaller and/or larger genetic changes (Peter et al., 2018). Small variations can be caused from single nucleotide and frame shift mutations, insertions or deletions, which will end up creating alterations in the structure/function of the encoding protein or alterations in the gene expression. Larger changes include structural variation such as chromosomal rearrangements (duplications, translocations, and inversions), segmental duplication and gene copy number variation. Genetic variations can also be driven from interspecific hybridization with introgression and horizontal gene transfer events. This enables the generation of novel characteristics to the genome that it could not occur with other nucleotide arrangements.

## Copy Number Variations and Re-Arrangements

Recent whole genome sequencing and large-scale phenotyping data for 157 *S. cerevisiae* industrial brewing strains revealed that these yeasts are genetically and phenotypically separated from their wild ancestors through complex domestication events in the man-made environments (Gallone et al., 2016). The analysis shows that industrial strains can tolerance various stresses, have better performance and lack sexual reproduction. Insertions and deletions of small or large fragments were detected in most of the strains analyzed with the size of the fragment varying from a few base pairs to complete chromosomes. Deletion patterns and copy number variations commonly found in beer strains are connected with the high aneuploidy or polyploidy characteristic of brewing strains. It was also observed that all the industrial strains even from different beer clades show clear marks of domestication and adaptation to industrial niches. This is consistent with the fact that the brewing industry is following common practices distinct from the wineries. In fact, wine yeast differs from the brewing ones, because of the seasonality of the wine production, the nutritional fluctuations and their sexual cycles. This can explain the population diversity (both in population size and in genome variety) of beer yeasts compared to wine yeasts. Chromosomal re-arrangements play an important role in the phenotypic variation of yeast strains both in the laboratory environment (Colson et al., 2004; Naseeb et al., 2016, 2017a) and in nature, where karyotypic instability is found in wild strains and can affect their performance when transferred to industrial settings (Carro et al., 2003; Dujon, 2010; Peter et al., 2018).

**FIGURE 1:**
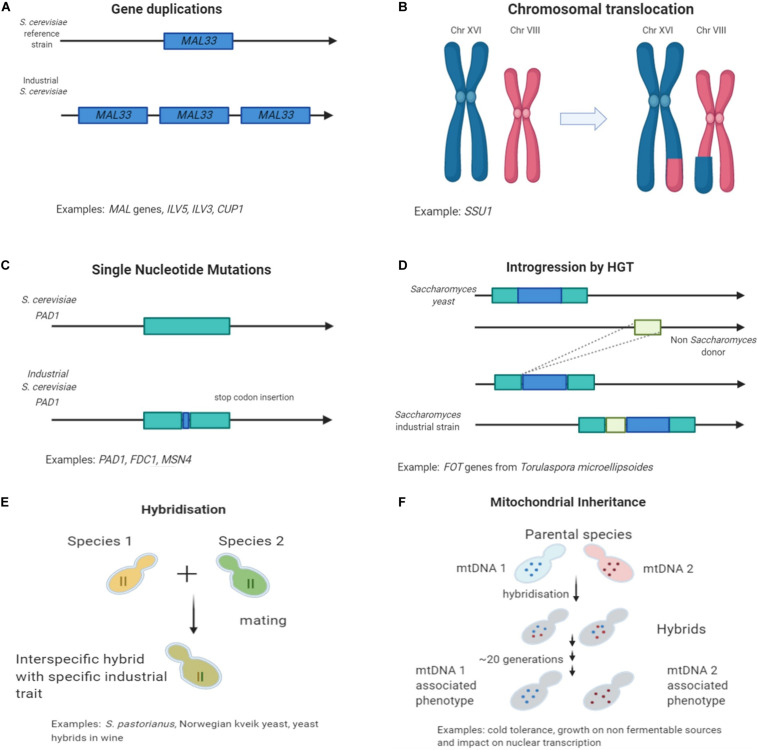
Mechanisms of genetic variation found in industrial *Saccharomyces* strains in fermentative environments. **(A)** Gene duplication and copy number variation; **(B)** Chromosomal re-arrangements; **(C)** Single nucleotide polymorphism causing loss of function; **(D)** Horizontal gene transfer events; **(E)** Interspecific hybridization; and **(F)** Mitochondrial inheritance.

Genome re-arrangements and copy number variations results also in extensive alterations of the gene expression network (Naseeb et al., 2016) and it is not only limited to the function of a specific duplicated gene. Any fitness improvement is resulting from both environmental and genomic conditions and multiple changes in the transcriptome (Guan et al., 2007; Hakes et al., 2007; Naseeb et al., 2017a). Furthermore, differences have been identified in the copy number of genes involved in the metabolism of fermentable sugars such as maltose and maltotriose. Copy number variations are often reported as an adaptation mechanism to environmental changes (Figure 1A; Magadum et al., 2013). The maltose metabolism consists of 3 gene families: *MALT*, *MALS*, and *MALR* which comprise maltose transporters, maltases and regulator proteins, respectively (Brown et al., 2010). Fluctuations in chromosomal location and copy number of the involved *MAL* genes are present in many industrial strains. Typically, beer strains contain six or more copies of the *MAL3* locus (Gonçalves et al., 2016). Some German yeast strains were found to contain 15 copies of the *MAL31* gene while wine *Saccharomyces* strains contains only three copies (Gonçalves et al., 2016). Uptake and breakdown of maltose, the main carbon source in beer fermentation but not in grape/must, has been of great importance for the survival and performance of brewing yeast strains. This remarkable genetic alteration in maltose uptake is giving a great selection of brewing candidates that are able of fast utilization of this sugar. van der Broek and co-workers examined chromosomal number variation in *S. pastorianus* and its link with the phenotype. They analyzed three *S. pastorianus* W34/70 isolates that produced different diacetyl concentrations during beer fermentation. Diacetyl, a vicinal diketone, with butter flavor is considered an undesirable metabolite occurring through yeast valine metabolism during beer fermentation (Kusunoki and Ogata, 2012). Analysis of the DNA sequence of the valine biosynthetic genes (*ILV2, ILV6, ILV5*, and *ILV3*) in the three isolates did not reveal any single nucleotide polymorphism, however, copy number variation in the chromosomes carrying those genes was identified. This resulted in one isolate containing extra copies of the *ILV5* and *ILV3* genes, that responsible for the downstream catalysis of a-acetolactate the precursor of diacetyl. Thus, this strain was a low-diacetyl producing isolate of W34/70 (van den Broek et al., 2015).

In wine yeast, studies have shown significant adaptation motifs to sulfite compounds. Sulfite contained chemicals are used extensively as preservatives in wineries. A way that yeast can tolerate the excess levels of sulfite is by increasing the regulation of the sulfite uptake and efflux through the *SSU1* plasma membrane pump encoded gene (Pérez-Ortín et al., 2002). An increase in expression of *SSU1* was observed in wine yeast strains compared to laboratory strains. Studies about the mechanism of sulfite resistance, identified a chromosomal translocation (Figure 1B) and non-homologous recombination of the *SSU1* gene promoter (Pérez-Ortín et al., 2002). Moreover, a chromosomal inversion between XVI and VIII connected with the *SSU1* regulatory region also result in overexpression of *SSU1* and thus in sulfite resistance to commercial wine strains (García-Ríos et al., 2019b).

Another adaptation in wine yeast has been triggered by the copper-based pesticides used in wineries. The *CUP1* gene, responsible for copper binding and mediating resistance to high concentrations, was found in a higher copy number in wine strains associated with higher resistance to CuSO_4_ compared with natural isolates (Almeida et al., 2015). Liu et al. (2015) identified a promoter variant of *CUP1* gene with increased expression suggesting that this benefit is involved in an adapting mechanism of the strains into a stressful condition. Interestingly, in organic vineyards where the usage of pesticides is strictly limited, a lower number of different yeast strains has been detected, as other wild micro-organisms naturally resistant to copper such as *Aureobasidium pullulans* and *Starmerella bacillaris* dominate (Grangeteau et al., 2017).

## Single Nucleotide Polymorphisms

Yeast domestication studies for beer and wine using sequencing technologies have unravel traits and performance improvements in different populations. In beer, an example illustrating well the process of trait improvement through selection and domestication, is the loss of function of genes related with ferulic acid decarboxylation. 4-vinylguaiacol is a phenolic compound with a distinct clove-like aroma. The decarboxylation of ferulic acid to 4-vinylguaiacol is occurring through yeast metabolism under the regulation of genes *PAD1* (phenylacrylic acid decarboxylase) and *FDC1* (ferulic acid decarboxylase) (Gallone et al., 2016; Gonçalves et al., 2016). The production of 4-vinylguaiacol during beer fermentation is considered a phenolic off-flavor (POF), and the strains are described as POF+. The clove-like aroma is considered characteristic only in some specific style of beers, such as Belgian and German wheat beers, but even for those a low threshold of POF would be desirable (Scholtes et al., 2014). Yeasts used for the production of alcoholic beverages produce an amount of undesired metabolites characterized as off-flavors and ideally that accumulation should be limited. The biological role of *PAD1* and *FDC1* is to help detoxifying phenylacrylic acids from the cell walls of plants (Mukai et al., 2014), which explains why wild yeast express those genes in order to survive and proliferate in natural habitats. Genomic studies revealed that in many industrial brewing strains the genes appear to be inactive and have acquired a frameshift mutation or a premature stop codon in the *PAD1* gene sequence (Figure 1C; Mukai et al., 2014; Chen et al., 2015). Interestingly, these type of mutations are not present in strains used in German like wheat beers. Such data show that different strains acquired different disruptive mutations, related to the presence of varied adaptive strategies in response to human selection against production of the POF+ character (Gallone et al., 2016).

Adaptation mechanisms due to different stressful conditions are also found yeast used in sake fermentation. These strains belong to the *Saccharomyces cerevisiae Kyokai* no. 7 group (K7). In sake brewing, the final ethanol content reaches almost 20%. Therefore, sake yeast genome has evolved to produce and accumulate high ethanol concentrations. The *Kyokai* strains express high fermentation rates. Both rapid fermentation and high ethanol production has been linked with environmental stress responses (Zhao and Bai, 2009). Studies in K7 yeast revealed a loss of function mutation in the genes *MSN4*, and *MSN2* that are responsible for transcription factors regulation during different types of stresses. Interestingly, the K7 group acquired a dysfunctional *MSN4* genes and results in high initial fermentation rate despite its lower stress tolerance compared to reference laboratory strains (Urbanczyk et al., 2011; Watanabe et al., 2011). The variant Km67 strain, belonging to the K7 sake group, has also been recently studied for its distinct characteristic of stress tolerance among the group. This strain has been used extensively and repeatedly as a starter culture for sake fermentation and surprisingly it doesn’t acquire the same loss-of-function mutation in stress response related genes and also confers unique sensory characteristics and high production of ethanol as the rest of the *Kyokai* group. This suggests that other underlying genomic adaptations have contributed to the phenotype and performance of Km67 and the strain represents a genetically distinguished isolate within the group (Takao et al., 2018). The same strain was also recently reported for high folate production compared to other strains in the K7 group but the mechanisms underlying this accumulation are yet to be determined (Shibata et al., 2019).

## Horizontal Gene Transfer

Horizontal gene transfer (HGT), including introgression of DNA fragments from one species to another, is a known mechanism to generate variation in prokaryotes and eukaryotes (Keeling and Palmer, 2008). In yeast, HGT has been proposed as a mechanism of genetic adaptation to a particular niche (Hall et al., 2005). For example, a wine yeast *S. cerevisiae* strain gained 2 *FOT* genes –responsible for encoding oligopeptide transporters- from *Torulaspora microellipsoides* by several HGT and re-arrangement events (Figure 1D). Gaining these genes conferred *Saccharomyces* a competitive advantage during wine fermentation as the strain could utilize more nitrogen sources and oligopeptides, enabling its cell viability and proliferation. As mentioned before, grape juice is often a nitrogen-limited environment thus the wine yeast are challenged from the availability (Marsit et al., 2015). In another study, in the wine strain *S. cerevisiae* EC1118, a unique gene has been identified contributing to glucose and fructose metabolism and adaptation to low nitrogen conditions. The genes were acquired from non *S. cerevisiae* donors mostly closely related to *Zygosaccharomyces rouxii* a wild species commonly found in wineries (Novo et al., 2009). HGT is a mechanism of acquisition of new genetic material between different species, not yet fully explored in eukaryotes, and further future studies will help understanding the causes, the likelihood and the environmental background of this genetic exchange.

## Hybridization

Another mechanism, that has facilitated the evolution of industrial species, is the generation of interspecific hybrids (Figure 1E). The novel resulting combinations of genes (Nakao et al., 2009; Hewitt et al., 2014) and proteins (Piatkowska et al., 2013)is contributing to unique characteristics and advantages for the progenies compared to the parental strains. These unique phenotypes enable survival and proliferation in a new environment with a better performance over the parental species.

Interspecific hybridization has been extensively studied in the *Saccharomyces*. The lager yeast *S. pastorianus*, is a hybrid between *S. cerevisiae and S. eubayanus* and it is the most widely known and used in the beer industry. The history of *S. pastorianus* can be tied to lager beer brewing during the winter months requiring a cooler fermentation temperature. A hybridization between *S. cerevisiae*, a great fermenter strain, and *S. eubayanus*, a cryotolerant isolate, created a new yeast suitable for adapting and performing in the new demanding of the beer making (Martini and Martini, 1987; Nakao et al., 2009; Hewitt et al., 2014; Monerawela and Bond, 2018). Several other hybrids have been isolated from industrial applications such as, hybrids of *S. cerevisiae* and *S. kudriavzevii* in beer, cider and wine (Masneuf et al., 1998; González et al., 2008) and hybrids of *S. cerevisiae* and *S. uvarum* (Le Jeune et al., 2007). The molecular drivers and biochemical pathways important for the cryotolerance trait in *S. kudriavzevii* has been recently unveiled, showing that temperature-induced redox imbalances could be compensated by either increased glycerol accumulation or production of cytosolic acetaldehyde (Paget et al., 2014).

Recently, a *S. cerevisiae* × *S. uvarum* unique hybrid was isolated from the Norwegian kveik farmhouse yeast (Krogerus et al., 2018b). Sequencing and phenotypic investigation on the strain showed that, this hybrid has been generated in brewing conditions and results in desirable characteristics such as tolerance to a wide temperature range, tolerance to high ethanol and good production of ester-flavor compounds.

Hybridization events have also been reported in wine strains. Garcia-Rios and co-workers constructed a non-GMO *S. cerevisiae* x *S. uvarum* hybrid to improve the wine fermentation properties of the parental *S. cerevisiae* strain. They performed growth and fermentation tests in a variety of different temperatures and media to evaluate the cryotolerance character of the hybrid. It is know that wine fermentations in colder temperatures improve the character, quality and fruit-flavor of wine (Molina et al., 2007). The hybrids generated were evaluated and compared to their parental strains in competition experiments to generate phenotypic maps and identify the different recombination events from the expressed phenotypes (García-Ríos et al., 2019a). Wine hybrids were constructed in order to generate strains able to survive and proliferate in low nitrogen levels commonly found in grapes (Su et al., 2019). Nitrogen is essential for yeast metabolism and fermentation ability as it is also responsible for the accumulation of aroma-related compounds (Rollero et al., 2018). Occurrence of natural interspecific triploid hybrids is also found in fermentative environments: *S. cerevisiae × S. cerevisiae × S. kudriavzevii* hybrids have been isolated from wineries providing a growth advantage in cold temperatures and high production of volatile thiols (Borneman et al., 2012). Triploid and tetraploid hybrids are also common in beer, with *S. pastorianus* forming two groups based on its DNA content. Saaz (Group 1) lager yeast are allotriploid strains with fermentation phenotypic characteristic close to the *S. eubayanus* parent while the Frohberg strains are allotetraploid with a similar fermentation performance to the *S. cerevisiae* parent (Walther et al., 2014). Hybrids generation among the *Saccharomyces* species have therefore resulted in a variety of phenotypes with great industrial fermentative potential (Bellon et al., 2011). Stress responses associated with wine and beer fermentation seem to have influenced the spontaneous generation of natural hybrids with different physiological traits.

## Mitochondrial Inheritance

Another important factor related to the occurrence of specific characteristics in industrial strains is the inheritance of mitochondrial DNA (mtDNA). During hybridization, different hybrids can inherit the mitochondria from either one or the other parental species (Figure 1F).

Compared to the nuclear genomes, the mtDNA in *Saccharomyces cerevisiae* is more diverted and highly assorted. Structural rearrangements are rare in mtDNA resulting in few cases of DNA loss (De Chiara et al., 2020), however, mitochondrial recombination is common and can lead to phenotypic differentiation if enough divergence is present in the parental species (Leducq et al., 2017). Recent studies showed a strong influence on the different parental mtDNA in *S. pastorianus* strains related with adaptation to cold temperatures (Baker et al., 2019; Hewitt et al., 2020). Yeast mitochondria contribute to evolutionary divergence of cold tolerant strains (Li et al., 2019) and the type of mtDNA inherited in the hybrids affects both the cellular fitness in different nutritional conditions (Albertin et al., 2013; Hewitt et al., 2020), and the nuclear transcription in the hybrid (Hewitt et al., 2020).

## Future Perspectives on Industrial Strain Improvement

The strong impact of molecular techniques and sequencing technologies have shed a light into evolutionary insights of industrial *Saccharomyces* species. Yeast domestication in man-made environments have been driven due to different stimuli that can include temperature and nutrient stresses, microbial competition, and ethanol and CO_2_ toxicity. Evidence of yeast adaptation in the fermentative environments show that an incredible variety of mechanisms, such as gene duplication events, chromosomal rearrangement, hybridization, HGT, and type of mitochondria inherited, contribute to re-shape the yeast genome for better survival traits.

Such acquired knowledge on yeast biology and evolution can now enable researchers to work on strain improvement and generate candidates that will facilitate the food and beverages industry. This include approaches such as selection of isolates with desirable characteristics, usage of non-conventional yeast and generation of hybrids.

Adaptive laboratory evolution experiments have introduced specific mutations in relevant traits or aneuploidy in domesticated yeast (Gorter De Vries et al., 2019), improved fermentation performance in polyploid strains and hybrids (Voordeckers et al., 2015; Krogerus et al., 2018a) and osmotic stress response resulted in shorter fermentation times (Ekberg et al., 2013). Laboratory adaptive evolution can lead to identification and observation of important fermentation characteristics as well as the ability to design and perform future experiments that will lead to yeast strain with desired industrial properties (Iattici et al., 2020).

Evolutionary selection is still the methodology of choice for yeast strain improvement and it’s usually preferred to classical breeding techniques. Breeding can generate progeny with desired phenotypic traits but is much more challenging because of yeast aneuploidy and poor sporulation efficiencies (Codon et al., 1995). The phenotypic characterization and evaluation of new strains improved via breeding can help the selection superior segregants that could be implemented in food and beverages industry (Sanchez et al., 2012; Figueiredo et al., 2017).

Interspecific hybrid sterility is a drawback in the generation of offspring with different combination of desirable traits. Several works have tried to overcome sterility through allotetraploidization. Through this method we are able to obtain fertile diploid spores, from an allotetraploid, allowing a recombination of traits between the different species (Greig et al., 2002; Sebastiani et al., 2002). The construction of complex *de novo* interspecific hybrids strains can result in phenotypic traits that weaken or strengthen under meiotic recombination and increase the diversity of the existing industrial candidates (Krogerus et al., 2017; Peris et al., 2020).

The fermentative potential of *Saccharomyces* species has not yet fully explored. *S. kudriavzevii* and *S. uvarum* are cold tolerant strains that have been isolated from fermentative environments. *S. mikatae*, *S. paradoxus*, *S. jurei*, yeast that possess traits such as cold tolerance and maltose utilization could be further exploited through interspecific hybridization (Fleet, 2006; Salvadó et al., 2011; Naseeb et al., 2017b, 2018). Hybrid strains can also address the need of aromatic novelty in fermented beverages (Nikulin et al., 2018).

Furthermore, applying population genomic studies will facilitate exploring the biodiversity of non-conventional yeast such as *Brettanomyces bruxellensis*, *Torulaspora delbrueckii*. These species, commonly found in wine and beer fermentations and characterized as spoilage yeast, have increasingly gained scientific and biotechnological interest (Avramova et al., 2018; Zhang et al., 2018). The occurrence and industrial potential of non-*Saccharomyces* yeast should further be considered as it will diversify the industrial strains suitable for generation of novel food products (Basso et al., 2016). In addition, the usage of mixed starter cultures of *Saccharomyces* and non-*Saccharomyces* strains can guide new product development with distinct flavors and performance characteristics. The exo-metabolites resulting from co-culturing strains during fermentation can influence yeast-yeast interactions and ultimately alter the population structure due to different competitive pressure (Ye et al., 2014).

Although there is now clear evidence for patterns of evolution and adaptation of *Saccharomyces* strains in the fermentative environments, not much is yet known on the survival of wild strains in natural habitats. Adaptation trajectories and mutations arising from extreme environmental conditions are still yet to be explored in wild yeast isolates (Aouizerat et al., 2019). Further exploration on the effect of harsh conditions and climate fluctuations on different yeast strains and species will broaden the understanding on how to maintain their biodiversity and on the importance of yeast-environment interactions.

## Author Contributions

KG performed the literature search with the inputs of DD and MC. KG and DD wrote the manuscript with the input of MC. All authors contributed to the article and approved the submitted version.

## Conflict of Interest

MC was employed by the company Cloudwater Brew Co. The remaining authors declare that the research was conducted in the absence of any commercial or financial relationships that could be construed as a potential conflict of interest.
